# DNA Barcoding of Sigmodontine Rodents: Identifying Wildlife Reservoirs of Zoonoses 

**DOI:** 10.1371/journal.pone.0080282

**Published:** 2013-11-11

**Authors:** Lívia Müller, Gislene L. Gonçalves, Pedro Cordeiro-Estrela, Jorge R. Marinho, Sérgio L. Althoff, André. F. Testoni, Enrique M. González, Thales R. O. Freitas

**Affiliations:** 1 Laboratório de Citogenética e Evolução, Departamento de Genética, Universidade Federal do Rio Grande do Sul, Porto Alegre, Rio Grande do Sul, Brazil; 2 Programa de Pós-Graduação em Genética e Biologia Molecular, Universidade Federal do Rio Grande do Sul, Porto Alegre, Rio Grande do Sul, Brazil; 3 Laboratório de Biologia e Parasitologia de Mamíferos Silvestres Reservatórios, Instituto Oswaldo Cruz, Fundação Oswaldo Cruz. Pavilhão Lauro Travassos, Rio de Janeiro, Rio de Janeiro, Brazil; 4 Laboratório de Mamíferos, Departamento de Sistemática e Ecologia, Universidade Federal da Paraíba, João Pessoa, Paraíba, Brazil; 5 Universidade Regional Integrada do Alto Uruguai e das Missões, Erechim, Rio Grande do Sul, Brazil; 6 Fundação Universidade Regional de Blumenau, Blumenau, Santa Catarina, Brazil; 7 Museo Nacional de Historia Natural, Montevideo, Uruguay; Institute of Biochemistry and Biology, Germany

## Abstract

Species identification through DNA barcoding is a tool to be added to taxonomic procedures, once it has been validated. Applying barcoding techniques in public health would aid in the identification and correct delimitation of the distribution of rodents from the subfamily Sigmodontinae. These rodents are reservoirs of etiological agents of zoonoses including arenaviruses, hantaviruses, Chagas disease and leishmaniasis. In this study we compared distance-based and probabilistic phylogenetic inference methods to evaluate the performance of cytochrome *c* oxidase subunit I (COI) in sigmodontine identification. A total of 130 sequences from 21 field-trapped species (13 genera), mainly from southern Brazil, were generated and analyzed, together with 58 GenBank sequences (24 species; 10 genera). Preliminary analysis revealed a 9.5% rate of misidentifications in the field, mainly of juveniles, which were reclassified after examination of external morphological characters and chromosome numbers. Distance and model-based methods of tree reconstruction retrieved similar topologies and monophyly for most species. Kernel density estimation of the distance distribution showed a clear barcoding gap with overlapping of intraspecific and interspecific densities < 1% and 21 species with mean intraspecific distance < 2%. Five species that are reservoirs of hantaviruses could be identified through DNA barcodes. Additionally, we provide information for the description of a putative new species, as well as the first COI sequence of the recently described genus *Drymoreomys*. The data also indicated an expansion of the distribution of *Calomys tener*. We emphasize that DNA barcoding should be used in combination with other taxonomic and systematic procedures in an integrative framework and based on properly identified museum collections, to improve identification procedures, especially in epidemiological surveillance and ecological assessments.

## Introduction

 Molecular identification of pathogens, their vectors, and their reservoirs is one of the expected applications of the DNA barcoding initiative. Knowledge of the exact species that is (are) carrying harmful pathogens is essential to studies of the factors leading to occurrences [1,2], pathogen proliferation [3], and transmission between animal vectors [4–6], as well as for epidemiological inferences as a whole. 

Worldwide initiatives such as the International Barcode of Life (iBOL) project, taxon-specific projects, and national funding programs have fostered the accumulation of massive standardized sequence data for the cytochrome oxidase *c* gene (COI), which has contributed significantly to species identification [7-12]. Both taxonomy and systematics have benefited from the Barcode of Life Database (BOLD), which integrates worldwide and species-wide sampling. This information source enables reliable identification of doubtful specimens only if the specimens in this database are correctly identified and named. Even though BOLD includes many species of metazoans, the identification process constitutes a taxonomic bottleneck [13]. In biodiversity hotspots where DNA barcoding would be most beneficial, there is a lack of scientific collections, taxonomists, and funding, particularly in the Atlantic Forest, Cerrado, Caatinga and Pampas biomes in Brazil. Thus, our major goal in this study was to investigate whether the DNA barcoding approach (hereafter barcoding) is a reliable tool for species identification of members of the speciose rodent subfamily Sigmodontinae, particularly in Brazil, where these rodents are hosts or form part of the reservoir system of several zoonotic diseases [2,5,6,14]. The success of barcoding as a species identification tool, as originally envisioned, rests primarily upon the performance of distance-based methods which depend on the existence of a disjunction between intra- and interspecific pairwise distance distributions, also known as the ‘barcoding gap’ [15] (but see 10,16,17 for alternative methods and [18] for an automated distance-based method). If a barcoding gap exists, empirical criteria, distance threshold values, or cutoff values could be used to define species limits [8], or at least, more empirically to sort specimens that merit detailed analysis [19] or as Primary Species Hypothesis to be tested [20] in large-scale inventories or epidemiological surveys. Although this approach is by no means a substitute for accurate taxonomic identifications [21], ecological studies or investigations of disease outbreaks must often evaluate both dominant species, which are abundant, and rare species that require careful taxonomic analysis and that will appear as outliers in distance-based analyses or related methods [22]. However, the extent to which this approach is useful in specific taxonomic groups is a matter for empirical evaluation.

 Sigmodontinae is the most diverse family-level mammalian clade in the Neotropical region [23,24] with up to 377 species [25]. These rodents are also important reservoirs of human diseases such as hemorrhagic fevers caused by arenaviruses [26] or hantaviruses [27], the etiological agents of hantavirus pulmonary syndrome (HPS) in the Americas. Hantaviruses are often associated with a single species [28] (but see [14,29]). At least 23 species (Sigmodontinae) are known to be hosts of hantaviruses, and six of these species were included in this study: *Oligoryzomys nigripes*, *O. flavescens*, *Necromys lasiurus*, *Akodon montensis*, *A. paranaensis* and *A. azarae*

Taxonomic studies in sigmodontine rodents are particularly dynamic, with new species constantly being described [30-36]. Also, recent large-scale taxonomic revisions have reshaped our understanding of the systematics of this group [23,37]. Molecular markers allow us to test taxonomic hypotheses, using mostly selectively neutral characters [23,38–41]. 

In this study, we examined the sequence variability of COI in sigmodontine species, particularly from southern Brazil, where most of the HPS cases have been reported [28,42]. We also evaluated the existence of a barcoding gap between intra- and interspecific distances, and tested the monophyly of putative species. This information was used to infer the utility of barcoding in species identification by comparing the results with field identifications and information from external morphology and karyotypes. We combined this dataset with sequences deposited in GenBank. We list taxa that can be accurately identified through the DNA barcoding approach. We also evaluate the reasons that other taxa might not be correctly classified using COI, and appraise field misidentifications.

## Materials and Methods

### Sampling

 Rodents were caught and handled according to the recommendations of the American Society of Mammalogists for the use of wild mammals in research [43]. Permission for fieldwork was granted by the Brazilian Ministry of Environment - IBAMA/ICMBio permanent collection permits 14690-1; 15224-2; 11375-1; 168/2004-CGFAU/LIC; 237/2005-CGFAU/LIC and by the Fauna Department of the Ministry of Livestock, Agriculture and Fishing of Uruguay, res. 176/2012. Rodents were trapped using Tomahawk® (Tomahawk Live Traps, Tomahawk, WI, USA) and Sherman (Sherman® - H.B. Sherman Traps, Tallahassee, FL, USA) live traps placed on the ground along linear transects and baited with a mixture of peanut butter, bananas, oatmeal and sardines. Traps were visited daily, early in the morning to minimize the time that the rodents remained within them. Animals were anesthetized with an intramuscular injection of ketamine (10-30 mg/kg) combined with acepromazine (5-10 mg/kg) (proportion 9:1), and euthanized with an intracardiac injection of KCl. Field procedures with rodents were approved by the Ethics Committee on Animal Use of the Oswaldo Cruz Foundation, Rio de Janeiro (P033607). 

**Figure 1:**
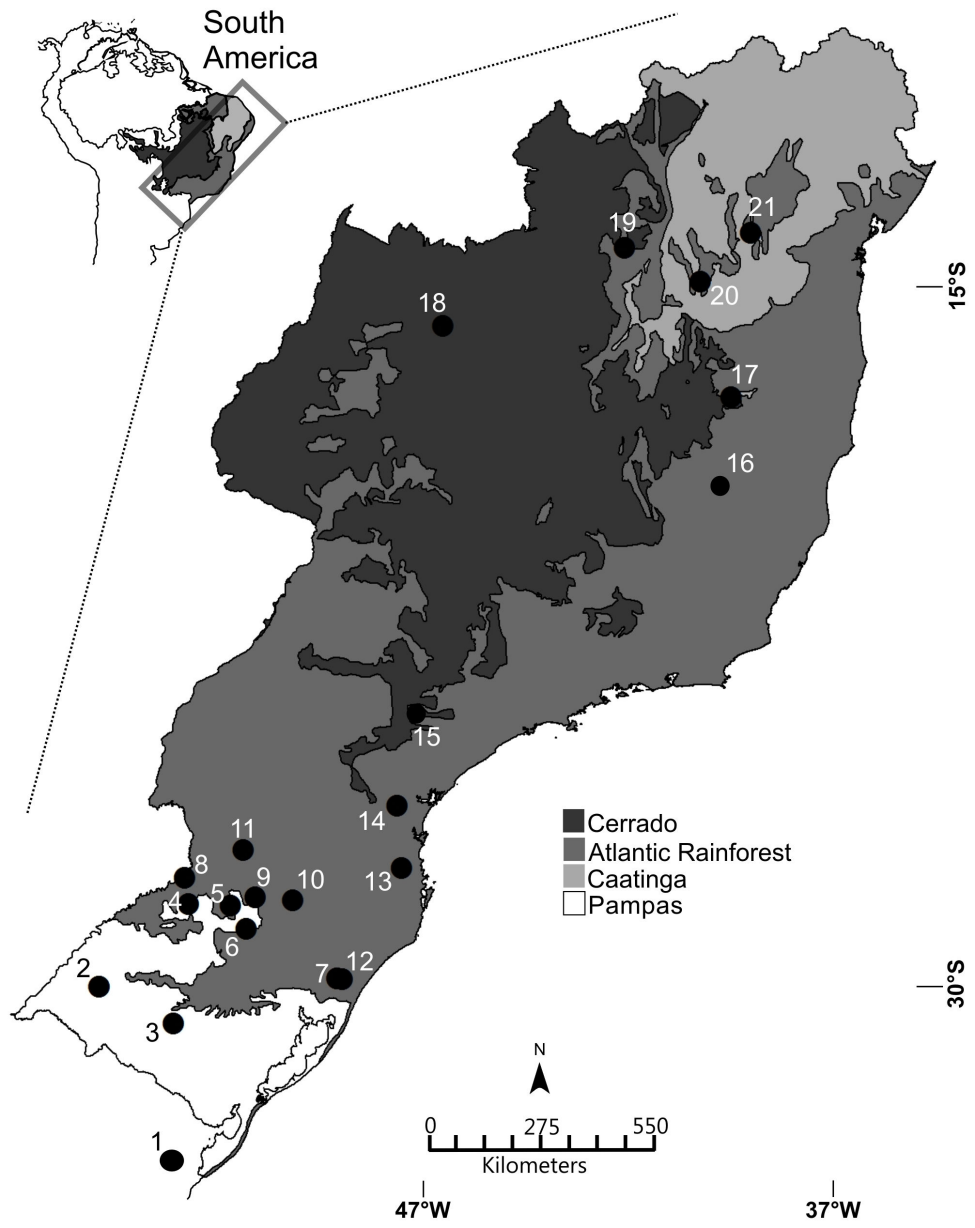
Biomes and localities sampled in South America. Different shades of gray correspond to different biomes, as indicated in the legend. 1. Reserva de Santa Teresa, Rocha, UY (-34.36; -53.46); 2. Alegrete, RS, BR (-55.71; -29.57); 3. Margarida do Sul, RS, BR (-54.07; -30.37); 4. Rondinha, RS, BR (-53.75; -27.75); 5. Ronda Alta, RS, BR (-52.82; -27.79); 6. Passo Fundo, RS, BR (-52.48; -28.29); 7. Terra de Areia, RS, BR(-50.48; -29.39); 8. Derrubadas, RS, BR (-53.83; -27.17); 9. Erechim, RS, BR (-52.28; -27.60); 10. Barracão, RS, BR (-51.46; -27.67); 11. São Domingos, SC, BR (-52.54; -26.56) 12. São Francisco de Paula, RS, BR (-50.37; -29.40); 13. Blumenau, SC, BR (-49.07; -26.96) 14. São José dos Pinhais, PR, BR (-49.17; -25.58); 15. Capão Bonito, SP, BR (-24.06; -48.32); 16. Capitão Andrade, MG, BR (-19.06; -41.81) 17. Itinga, MG, BR (-41.82; -16.61) 18. Mimoso de Goiás, GO, BR (-15.06; -48.19); 19. Correntina, BA, BR (-44.16; -13.34); 20. Caetité, BA, BR (-42.50;-14.07); 21. Mucugê, BA, BR (-41.39; -13). BR: Brazil. UY: Uruguay. Brazilian States: RS-Rio Grande do Sul, SC-Santa Catarina, PR-Paraná, SP- São Paulo, MG-Minas Gerais, GO-Goiás, BA-Bahia. Coordinates in parentheses are given in decimal degrees.

A total of 130 sigmodontine specimens, including 13 genera and 21 species, were collected in 21 localities across the Cerrado, Caatinga, Pampas and Atlantic Forest biomes in Brazil (Figure 1, Table S1). Most samples (64%) have additional information, such as diploid number and vouchers deposited in collections. The species surveyed represent the major tribes: Oryzomyini, Akodontini, Thomasomyini and Phyllotini. Species with controversial allocation at the tribal level, including *Juliomys pictipes, Rhagomys rufescens, Delomys dorsalis* and *D. sublineatus*, were also analyzed. All sequences have been deposited in GenBank (accession numbers GU938873-GU939002) and BOLD under the project Rodents from Southern Brazil-ROSBR; Table S1). Sigmodontine sequences available in GenBank were also added in our analysis (N=58, 24 species): EU095420, EU095443-75, EU095488-93, EU096809-25, EU096953. This study is part of BrBOL (Brazilian Barcode of Life; http://www.brbol.org/), under the project Tetrapoda. 

### DNA extraction, PCR amplification and sequencing

Total genomic DNA was extracted from tissue samples using CTAB [44] and phenol chloroform [45] protocols. The target region of 648-bp COI was amplified through polymerase chain reaction (PCR) using the universal primer pair LCO1490 (5'-GGTCAACAAATCATAAAGATATTGG-3') and HCO2198 (5'-TAAACTTCAGGGTGACCAAAAAATCA-3') [46]. The PCR reaction included 1 U of Taq polymerase (Invitrogen) and 1.2 mM MgCl_2_, at 50°C of annealing temperature in a 20-µl reaction volume. PCR products were cleaned through the ExoSap (GE Healthcare) enzymatic method, sequenced with BigDye chemistry, and analyzed on an ABI3730XL (Applied Biosystems) at Macrogen® (Republic of Korea).

### Data analysis

 Chromatograms were edited in Chromas 2.4 (Technelysium Pty Ltd., South Brisbane, Australia) and the sequences were automatically aligned using Clustal X implemented in the software MEGA 5 [47]. We tested for substitution saturation in the dataset with the program DAMBE 5.2.61 [48] using Kimura 1980 (K80) and General Time-Reversible (GTR) models. Sequence divergence was calculated using the Kimura two-parameter (K2P) base substitution model [49]. We calculated divergence averages (mean and variance) at different taxonomic levels (intraspecific, interspecific, intrageneric and intertribal) from the pairwise distance matrix. Distance distributions were evaluated through histograms. The barcoding gap was evaluated by comparing the intraspecific and interspecific distance distribution assessed by kernel density estimation. The area of overlap of the density curves was calculated. Neighbor-joining (NJ) trees based on K2P distances were inferred to represent patterns of divergence among taxa, using the ape library [50] in R software [51].

Model selection of sequence evolution was estimated using MrAIC [52] with the BIC criterion. Phylogenetic reconstruction was carried out using Maximum likelihood (ML) and Bayesian inference (BI) because mitochondrial genes have been shown to have a phylogenetic signal in sigmodontines [39]. ML trees were obtained with PHYML 2.4.4 [53] and BI using MrBayes 3.1 [54] with 2 independent runs of 4 chains for 5,000,000 generations. Monophyly-confidence limits were assessed with the bootstrap method [55] at 50% cut-off after 1000 bootstrap iterations for ML, and posterior probabilities (pp) for the BI analysis. Distance-based and probabilistic trees were compared to evaluate the congruence of monophyletic groups. The neotomine species *Peromyscus maniculatus* (GenBank: EF568630) was used to root the tree in all analyses. Trees were plotted and edited using the software FigTree 1.2 [56].

### Specimen reclassification

Discrepancies between taxonomic identifications made in the field and phylogenetic placement in our analysis were solved using different lines of evidence considering the information available for each specimen, supported by previous taxonomic knowledge. First, we rechecked morphological characters, following an identification key for genera based on external characters [57] and examined skulls of specimens deposited in collections. Second, we paid particular attention to juvenile specimens since they are often misidentified, e.g., young specimens of *Delomys* are frequently misidentified as an adult of *Euryoryzomys*. Third, we examined the diploid number of specimens that were previously karyotyped, particularly for species of the genus *Akodon*, which have few or no clear-cut external discriminant characters, depending on the species pair (see [58] for a synthesis). Fourth, for samples with unavailable vouchers, we analyzed the external measurements taken during field work and evaluated the nestedness of specimens relative to specimens with available vouchers.

## Results

### Barcode divergences between species

COI sequences surveyed did not show saturation for either transitions or transversions (Figure S1). First, we performed a distance-based analysis of intraspecific and intrageneric genetic distances. A few discrepant values, i.e., intraspecific differences higher than expected (>10%), were observed. Second, we compared NJ distance-based trees to ML and BI phylogenies obtained from the same data set. All distance clusters were congruent with clades from ML and BI except for the topology among three genera. In the BI and ML inferences, *Calomys* and *Delomys* (pp = 1) were monophyletic, whereas in the NJ tree, *Calomys* grouped with *Rhagomys* (albeit with bootstrap < 50). 

**Figure 2:**
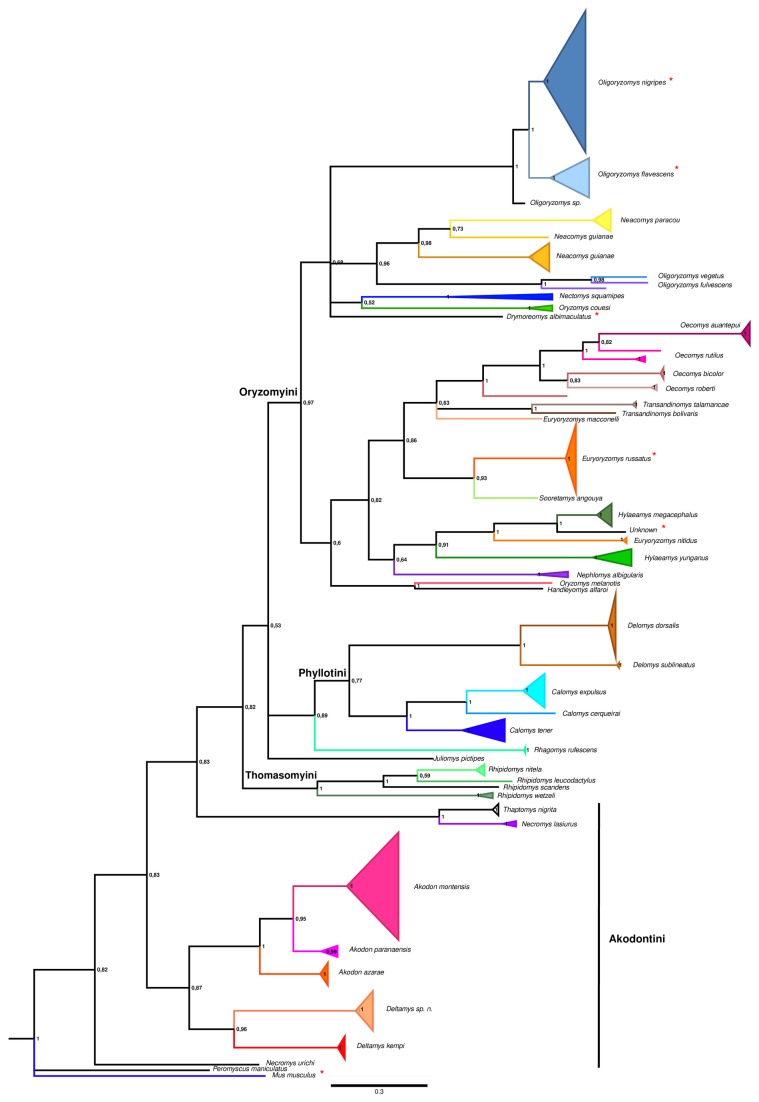
Bayesian phylogeny of Sigmodontine rodents inferred from 648 bp of COI. Species are depicted with branches of different colors. Triangle width is proportional to sample size (see Table S1), and depth to branching depth. Species misidentifications are marked with an asterisk. Tribal membership is indicated by the colored background. Posterior probability values are indicated on nodes.

Misidentified specimens are marked with an asterisk in Figure 2 (see below for discussion). They were reclassified either as a known species if the sequence was nested within a clade, or as monophyletic after rechecking external morphological characters (using an identification key for the genus level, Bonvicino et al. [2008] and karyotype data when available) or as an unknown taxon if it was not closely related to any other clade (K2P distance > 2%), which occurred for a single sequence. 

Descriptive statistics are given as a benchmark for subsequent studies using COI, since the characteristics of evolutionary distance distributions have been described only seldom. Pairwise K2P distances among all specimens ranged from 0 to 29.3% (mean = 14.7%, variance [σ^2^] = 0.002) with a bimodal frequency distribution, one mode centered near 0% and the other around 15% (data not shown). Intraspecific variation ranged from 0 to 16.7% (mean = 2.4%; σ^2^ = 0.001). Intrageneric mean distance values varied between 11.2% and 19.7% (mean = 15.2%, σ^2^ = 0.0007). Intrageneric distances of *Deltamys, Handleyomys, Juliomys, Necromys, Nectomys, Nephlomys, Oxymycterus, Rhagomys, Scapteromys, Sooretamys*, and *Thaptomys* were not calculated since each was represented by only a single species in our sample.

*Oligoryzomys vegetus* (EU095457) and *O. fulvescens* (EU095455-56) were removed from the data set for these calculations, because they were paraphyletic to other *Oligoryzomys* spp. and showed a mean distance of 14.9% from other *Oligoryzomys*. We also removed *Necromys urichi* (EU095420) due to a highly discrepant phylogenetic position with regard to other members of the tribe Akodontini (mean = 15.4 %, σ^2^ =0.0001). The decision to remove these taxa was based on (i) their erratic phylogenetic position, (ii) large distances from other congeneric species, and (iii) our purpose of providing benchmark distances, which could be compromised by those specimens belonging to genera that are hantavirus reservoirs. Intertribal distances calculated among Akodontini, Oryzomyini, and the *incertae sedis* groups ranged from 9.6 to 14.6% (mean = 12.3%, σ^2^ = 0.0006). Histograms of each taxon showed a bimodal distribution; all of them showed one small mode centered at 0% and another mode around the mean (data not shown). 

**Figure 3:**
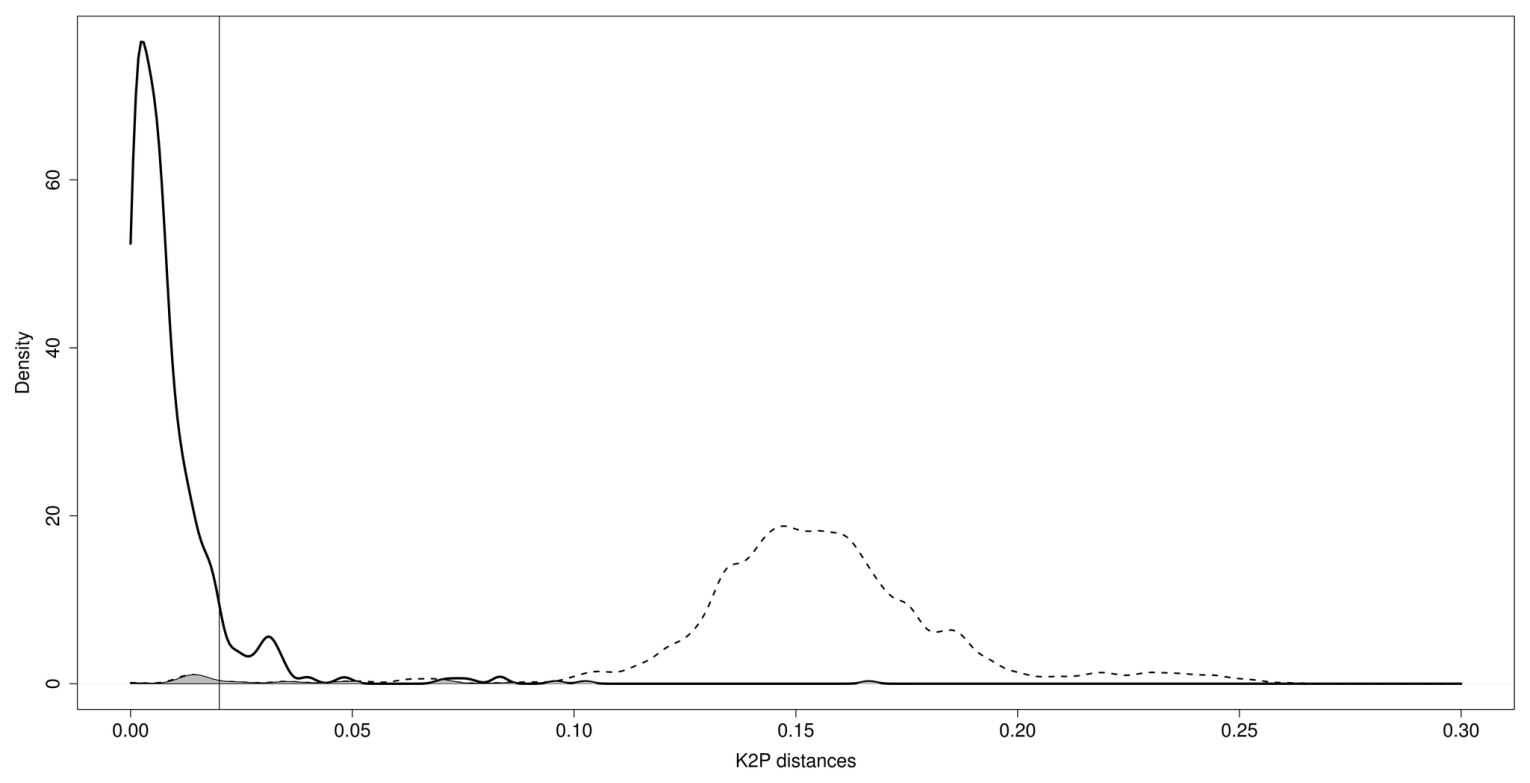
Intra- (solid line) and interspecific (dotted line) pairwise genetic distance (K2P) distribution. Area overlap between distributions (< 0.01) is shaded. The 2% threshold is indicated as a vertical line. Distributions are estimated by kernel density estimation.

**Figure 4:**
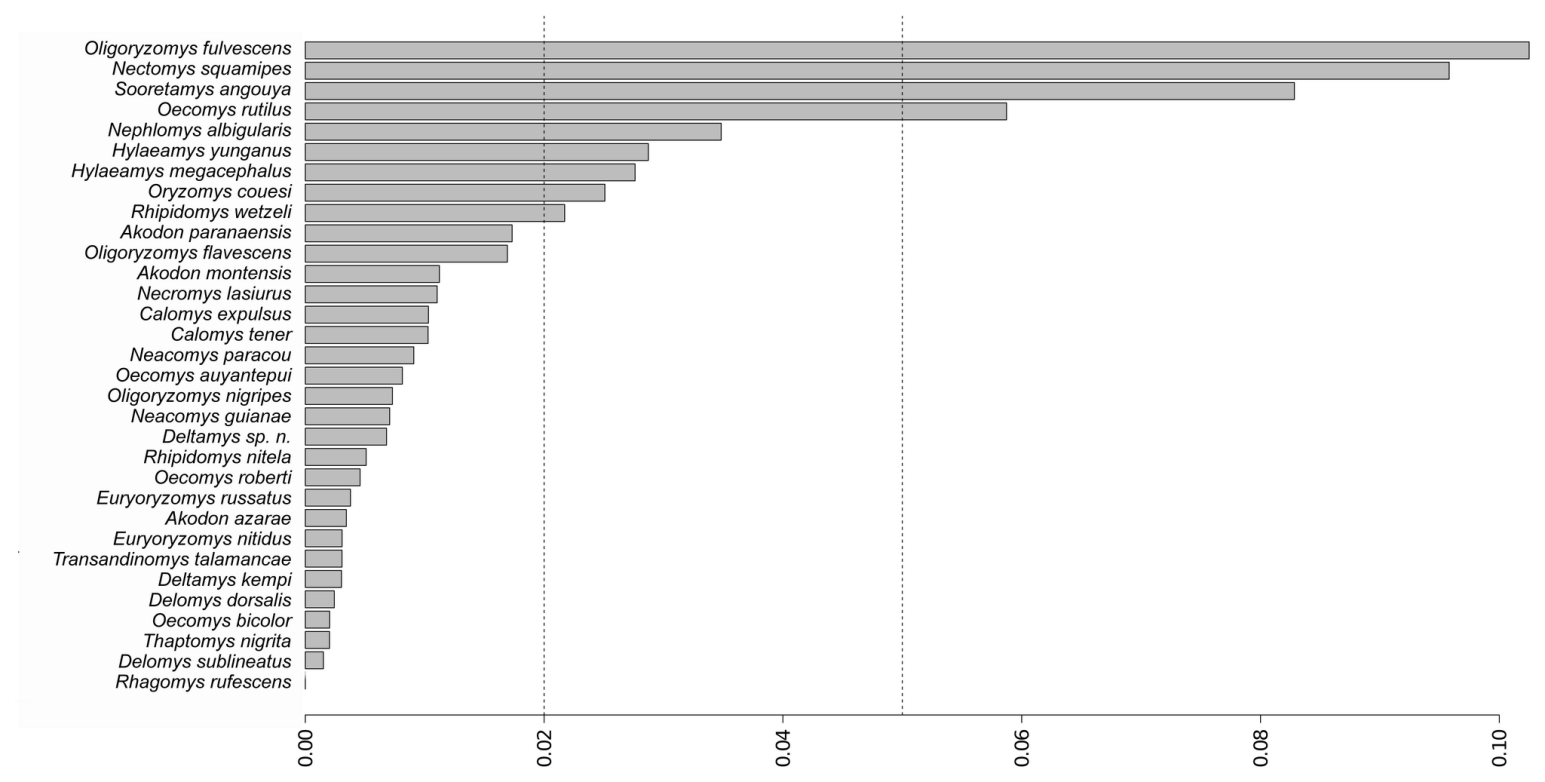
Mean intraspecific K2P pairwise distance after reclassification. First line indicates the 2% threshold of intraspecific variation. Twenty-three species are below this limit and nine are above. The second line indicates 5% genetic divergence.

The overlap between the areas of curves of the distribution of pairwise intraspecific (mean = 0.9%, σ^2^ = 0.0002) and interspecific (mean = 15.4%, σ^2^ = 0.001) distances was < 0.01, which numerically shows the existence of a clear barcoding gap (Figure 3). A total of 22 of 45 species showed mean intraspecific genetic distances < 2% (Figure 4). 

#### Phylogenetic analysis

 The results of the estimation of the proportion of invariant sites, transition/transversion ratios, nucleotide frequencies, gamma shape parameter and likelihood of ML and BI inferences are compared in Table S2. Likelihood scores stabilized after 1,000,000 generations in the Bayesian Inference. The Potential Scale Reduction Factor reached 1, indicating convergence between runs. A total of 490,000 samples were kept for summary statistics.

#### Barcode recovery

**Figure 5:**
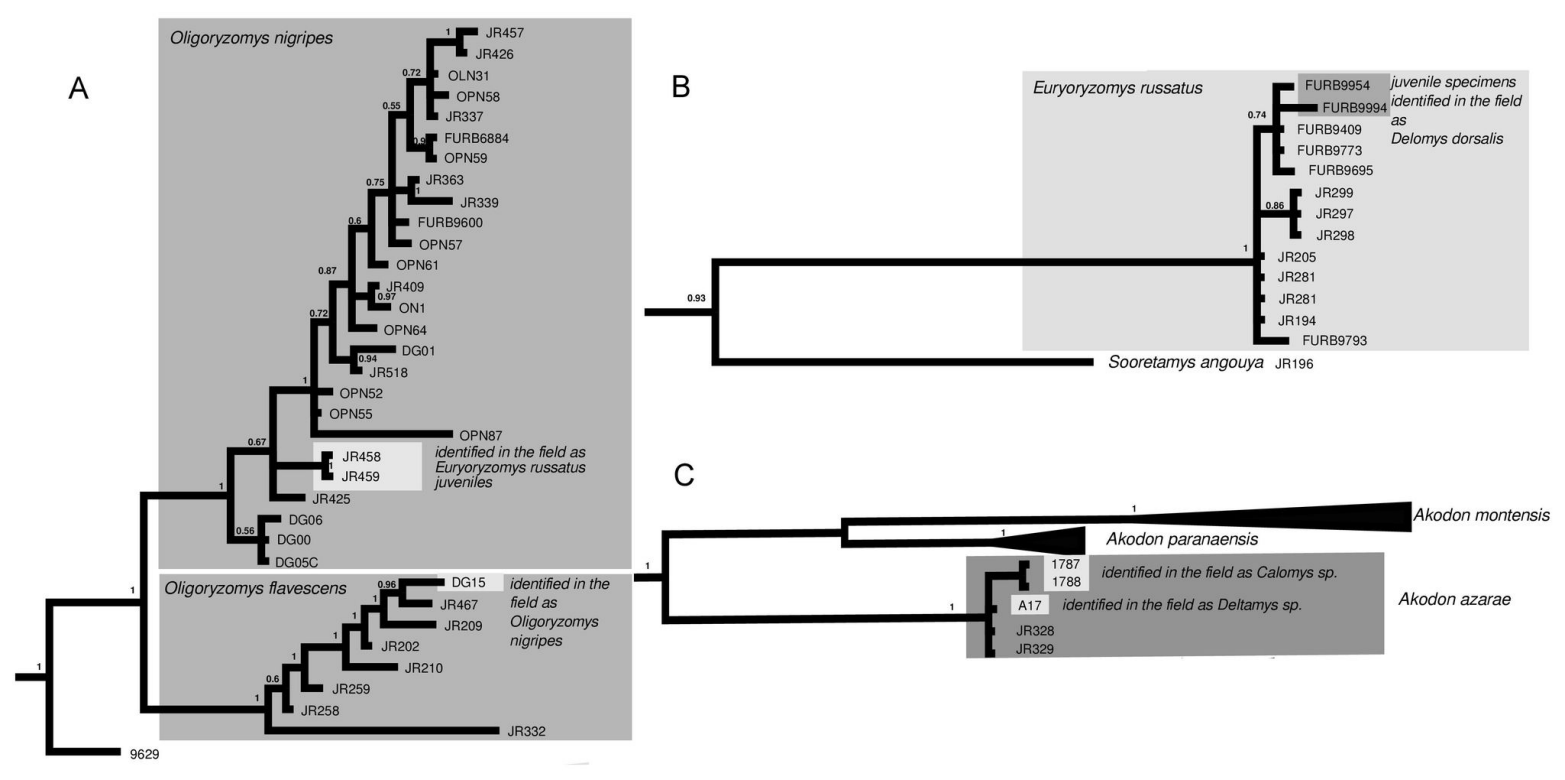
Detail of misidentified specimens. Subtrees shown in Figure 2 from the Bayesian phylogeny inferred based on 648 bp of COI for sigmodontine rodents (posterior probability values on nodes) are shown in detail. Misidentified specimens are indicated with a light background. A: subtree for *Oligoryzomys nigripes* and *O. flavescens*; B: subtree for *Euryoryzomys russatus*; C: subtree for *Akodon* species.

Similar topologies from NJ, BI and ML trees were retrieved, varying mainly in branch support values. Monophyletic groups were recovered for at least 41 of the 45 species analyzed and for 20 of the 25 genera. Most of the species were correctly recovered, with high branch support (Figure 2). Eight of 126 specimens clustered within unrelated clades (*Calomys* sp. 1787 and 1788, *Delomys dorsalis* FURB9954, *D. sublineatus* FURB9994, *Euryoryzomys russatus* JR458 and JR459, *Oligoryzomys nigripes* DG15 and sample 1007), indicating a conflict between the phylogenetic position and the field identifications (Figure 5). These samples were reclassified according to their phylogenetic placement. Two specimens of *Juliomys pictipes* did not cluster together; FURB9667 was identified by BLAST as *Mus musculus* and is likely a mislabeled sample. *Akodon* sp. PCE24 was monophyletic with other *Akodon montensis* sequences and showed a low K2P distance (< 2%) with other *A*. *montensis* (Table S1) Sample FURB9629 probably belongs to the genus *Oligoryzomys*. Although it clustered with other *Oligoryzomys* sequences, it was not nested within any of the species sampled. This example highlights the need for large individual taxon sampling for correct identification.

**Figure 6:**
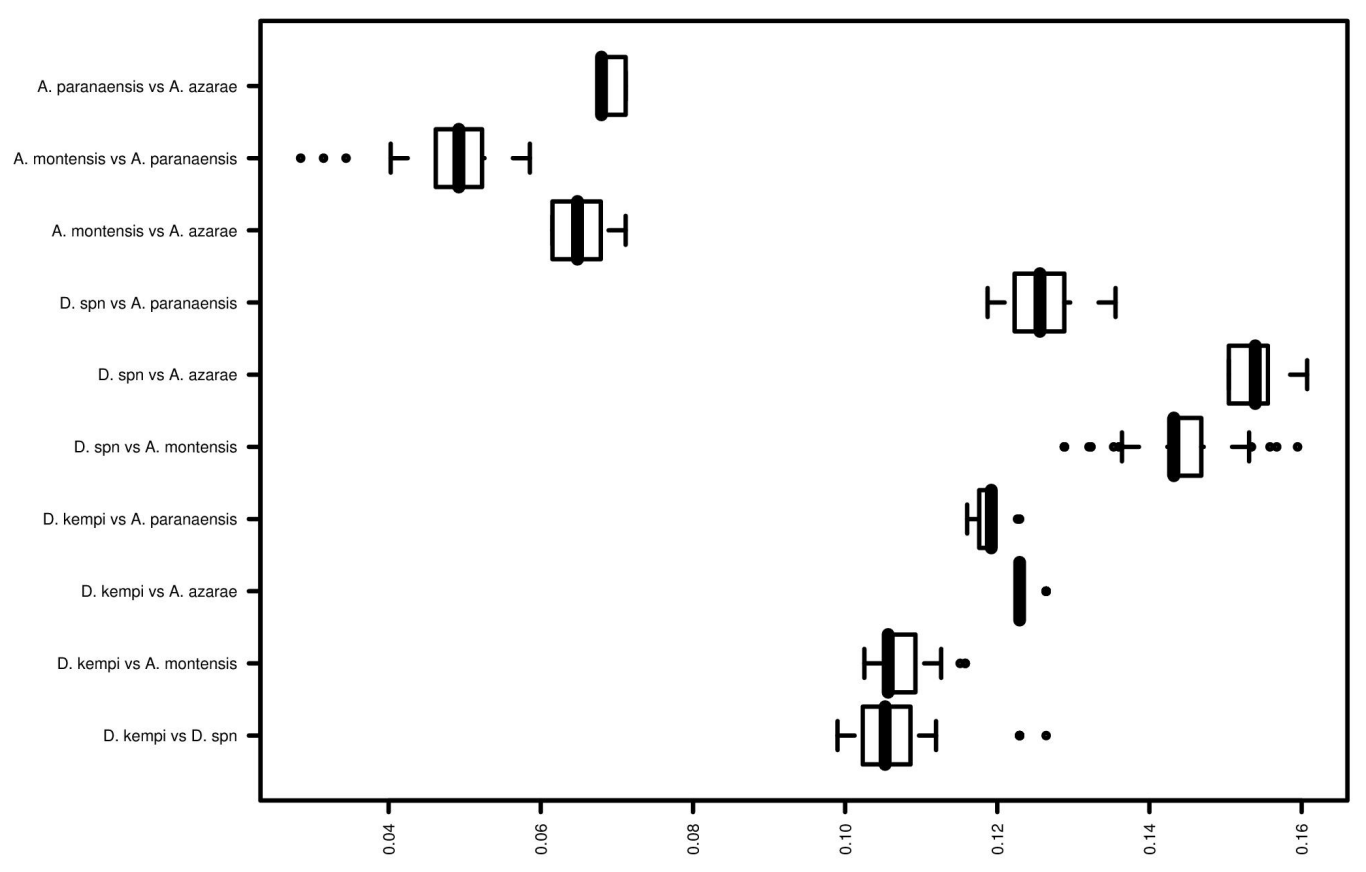
Box-and-whisker plot of evolutionary divergence among *Deltamys kempi*, *Deltamys* sp. n. and *Akodon* species. The box represents the median, first and third quartile, whiskers represent 1.5 times the interquantile range. Dots represent outliers. Distances were calculated based on Kimura 2-parameter model.

*Oecomys, Transandinomys*, and *Neacomys* were monophyletic, whereas *Akodon*, *Oligoryzomys, Euryoryzomys, Oryzomys*, and *Hylaeamys* were paraphyletic. All these paraphylies involved sequences from GenBank. The *incertae sedis* species *Delomys dorsalis* and *D. sublineatus* were monophyletic with *Calomys* (pp = 1). *Rhagomys rufescens* was monophyletic with *Delomys* and *Calomys* (pp = 0.93). The species of Akodontini were split into three monophyletic groups, one containing species of *Akodon* and *Deltamys*, another including *Necromys lasiurus* and *Thaptomys nigrita*, and a third group including *Necromys urichi*, which was the sister-group of all other sigmodontines. A group of sequences that we named *Deltamys* sp. n. was monophyletic and appeared as the sister-group of *D. kempi* (Figure 2). Interspecific pairwise distance comparisons suggest that this group likely represents an undescribed species of *Deltamys* (Figure 6).

## Discussion

### Identification of hantavirus reservoirs through barcoding

Most of the species and genera surveyed in this study, particularly those that are hantavirus reservoirs, were successfully identified through DNA barcodes. These taxa also included misidentified specimens. Identifying the taxa and sources of misidentifications is a critical step toward improving barcoding or identification practices. At least with respect to sigmodontine rodents and hantaviruses, instead of applying wide-ranging automated methods, taxon-specific discussions could improve our understanding of the potential pitfalls in identification procedures. This understanding should be applied toward improvements in identification procedures that would dramatically enhance the presently incomplete distribution maps, which is a major drawback to understanding the evolutionary history of hantaviruses [59] or the ecological determinants of transmission risk [60].

Correct assignment of *Oligoryzomys* species, for example, is critical because at least eight species within this genus are hantavirus reservoirs [2,27,59]. The genus includes around 18 species [2,25] and its taxonomy is very dynamic [2,61,62]. The complexity of the taxonomy, the lack of a clear phylogenetic framework, and the sympatry of several species make it difficult to determine the factors affecting the transmission of hantavirus [60]. *Oligoryzomys nigripes* is associated with the Juquitiba virus (JUQV) [28], and *O. flavescens* with the Lechiguanas virus. Two specimens initially identified by external morphology as *Euryoryzomys russatus* (JR458 and JR459) were monophyletic, together with 24 specimens of *Oligoryzomys nigripes*. Although the DNA of these samples was extracted from ear tissues gathered during ecological assessments and not from museum vouchers, we assigned them to *O. nigripes*, based on the overall appearance of the rodents recorded in field notes (i.e., long hind feet, higher tail/body length ratio, very small body size). Juveniles of *E. russatus* are quite similar to *O. nigripes*. Specimen DG15, previously identified as *O. nigripes*, clustered together with *O. flavescens*, clearly indicating a field misidentification, which was undoubtedly due to the close similarity of external diagnostic characters [57].

 Species of *Akodon* are also of particular interest; *Akodon montensis* is associated with the Ape Aime-Itapúa virus (AAAI) in Paraguay (see [29,63]); the Jabora virus (JABV) has been identified in *A. montensis*, *A. paranaensis*, and *A. serrensis* [14,28,42]; and the Araucaria virus (JUQ-like virus) is found in *A. montensis* and *A. paranaensis* [42,64]. The high node support within *Akodon* strongly suggests that barcoding can be successfully used to identify species in this group.

### Misidentifications and the taxonomic bottleneck

 A few studies have investigated COI sequence divergence in Neotropical rodents. Borisenko et al. [65] investigated small mammals (opossums, bats and rodents) from Guiana and Suriname and suggested the effectiveness of this target region in species identification. Our data substantially increased the taxonomic and geographical sampling, and showed that interspecific and intraspecific variations have isolated distributions to such an extent that a gap exists, which enables unidentified individuals to be assigned to their species with a negligible error rate (< 1% based on the overlap of inter- and intraspecific-distance distributions). This gap was not congruent with a previously proposed 2% species limit [8]; instead, a 5% threshold would be more appropriate for this rodent subfamily. The analysis of distance distributions as estimated by kernel density estimators is an alternative method to sliding-windows methods [18]. The relative performance of these methods still needs to be tested.

 Most of the specimen identifications based on external characters were correct; these were mainly carried out by mammalogists with several years of field experience but without a strict taxonomic background. Additionally, some specimens were identified by undergraduate students during student projects. Misidentified specimens comprised 9.5% of the sample. Despite the large number of specimens of Sigmodontinae collected either for epidemiological studies or for ecological assessments, we are not aware of another estimate of misidentification percentages. This information could be valuable in both contexts, since epidemiological surveillance and construction permits are based on species lists. Here, we have shown that the presence of juveniles might increase misidentification percentages. As previously stated, juveniles of *E. russatus* were misidentified as *O. nigripes*. Yet another example of misidentification of juveniles in the field could be detected in 10 samples of *E. russatus* that were monophyletic with samples FURB 9954 and FURB 9994, identified in the field as *D. dorsalis* and *D. sublineatus*. These two specimens are more related to three specimens of *E. russatus* collected in the same locality. However, analysis of museum specimens FURB 9994 and FURB 9954 showed that they are juveniles, reinforcing our hypothesis of field misidentification. 

 Misidentifications were initially detected from the barcodes, through discrepancies in intraspecific distance ranges and/or phylogenetic position. When specimens clustered within unrelated taxonomic groups and their genetic distance from conspecifics was higher than expected based on other intraspecific values, we considered these specimens to be misidentified. We suggest that these are misidentifications rather than cases of incomplete lineage sorting, introgression, or current gene flow, particularly because the statuses of these species have been reasonably well established through previous taxonomic studies using integrative approaches such as cytogenetics, morphology, morphometrics, and geographical distribution [2,58,62]. 

 However, taxonomic expertise is necessary to check possible misidentifications. For example, one specific specimen (FURB9792) fell within the tribe Oryzomyni, but with low support. Further examination of morphological characters of the voucher confirmed that this specimen is *Drymoreomys albimaculatus*, a recently described species from mid-altitude Atlantic Forest of São Paulo and Santa Catarina states [35]. Interestingly, the closest genus to *Drymoreomys* is *Eremoryzomys polius*, which occurs in the Peruvian Andes. Although a few taxa have shown similar patterns [35,66], this point highlights the need for intense efforts to build DNA barcoding libraries for the identification of rare, endemic taxa with deep phylogenetic divergences. Furthermore, this example clearly shows that redoubled attention should be paid to those lineages that reveal unique taxa.

### Monitoring species ranges and species discovery

*Calomys tener* occurs in the Cerrado biome [38,57]. Surprisingly, one specimen sampled in our study was captured in Alegrete Municipality (Figure 1), a grassland area which is isolated from other collection localities in the Cerrado by a belt of Atlantic Forest. This finding indicates either an introduction of *C. tener* into the southern grasslands, or a past connection between the southern grasslands known as the Pampas biome and the Cerrado savanna-like grasslands. This latter hypothesis is reinforced by the finding of another specimen on the coastal plain of Rio Grande do Sul state, in Quintão Municipality [29°40’S, 50°12’W] [67]. Other researchers have found evidence of an introduction or range expansion of a second *Calomys* species in this region (*C. laucha*) [68]. These results highlight the potential use of barcoding to monitor species ranges. This issue is particularly sensitive as trade flows intensifies and agroecosystems expand rapidly in Brazil. A congeneric species, *C. musculinus*, which occurs in Argentina and is a known reservoir of Junin arenavirus, has shown substantial range expansion driven by agricultural expansion [69,70].

 The barcoding results also support the recognition of a new akodontine species. Previously assigned as *Akodon* sp. (E. Pedó, Personal communication), it was strongly recovered as the sister-group of *Deltamys kempi*, while all other *Akodon* species were monophyletic (Figure 2). Furthermore, the genetic distances among *Akodon* taxa are less than 8%; between *Deltamys kempi* and *Deltamys* sp. n. the genetic distance ranges from 10 to 11%; and between *Deltamys* sp. n. and the other *Akodon* species it ranges from from 12-16%. The diploid number of this putatively new species is 2n=34, whereas *D. kempi* has a multiple sex chromosome system with 2n= 35, 36, 37, 38 [71–73]. Recently, a new allopatric lineage of *Deltamys* was proposed [74], with a diploid number of 2n=40. *Akodon* and *Deltamys* belong to the same tribe (Akodontini), and are sister genera according to nuclear and mitochondrial genes [39]; they can be distinguished by a single small chromosome pair that is present only in *Akodon.*

These examples illustrate the potential of barcoding efforts to provide additional characters for the recognition of new species. Nevertheless, the formal description of species depends on the evidence of additional characters for taxa delimitation, evaluation of available names, and most importantly, analysis of type specimens. This taxonomic bottleneck could be partly overcome if initiatives were undertaken to barcode type specimens, which are the name bearers. These examples show two important points to be addressed. 

First, there is a large amount of material collected either by inexpert mammalogists or in projects not focused on taxonomy. This situation is often overlooked and/or understated in the literature, and has profound implications for epidemiological surveillance and biodiversity assessments. We emphasize that the limited number of taxonomists and proper infrastructure for natural history collections will be an important bottleneck for the success of barcoding as an identification tool. Identifying misclassifications requires background knowledge about the taxa distribution, phylogenetic position, and range of variation. Likewise, building solid sequence databases for identification, as iBOL is proposing, will require not only that these be developed with the close collaboration of taxonomists, but that they also include either holotype or topotype specimens that will establish valid names for the monophyletic groups or clusters analyzed.

## Supporting Information

Figure S1 **Saturation plots for K2P distances and GTR distances for transitions (s) and transversions (v).**
(TIFF)

Table S1 **List of specimens grouped by species and localities.**
(DOC)

Table S2 **Parameter estimates for GTR+I+G model from Maximum likelihood (ML) and Bayesian inference (BI).**
(DOC)
