# Supplementary figures and images for: DNA Barcoding of Sigmodontine Rodents: Identifying Wildlife Reservoirs of Zoonoses
Source: PLoS One. 2013 Nov 11;8(11):e80282. doi: 10.1371/journal.pone.0080282 (PMC3823626; doi:10.1371/journal.pone.0080282)

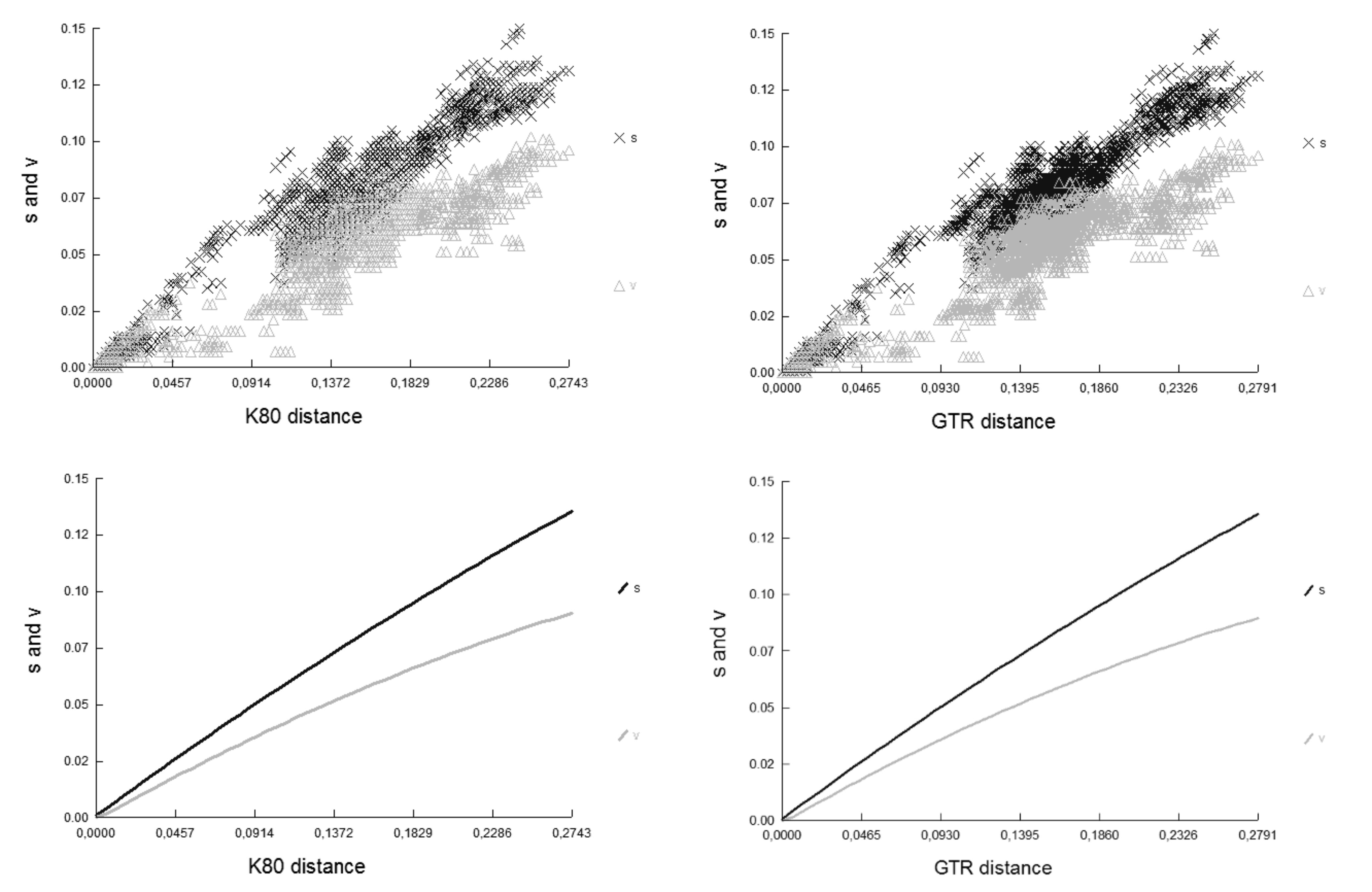

Supplement: Figure S1 — Saturation plots for K2P distances and GTR distances for transitions (s) and transversions (v). (TIFF) [file pone.0080282.s001.tiff]
