# Supplementary material for: DNA Barcoding of Sigmodontine Rodents: Identifying Wildlife Reservoirs of Zoonoses
Source: PLoS One. 2013 Nov 11;8(11):e80282. doi: 10.1371/journal.pone.0080282 (PMC3823626; doi:10.1371/journal.pone.0080282)
Supplement: Table S1 — List of specimens grouped by species and localities. (DOC) [file pone.0080282.s002.doc]

**Table S1.** List of specimens grouped by species and localities.

| **Sample ID** | **Field ID** | **GenBank** | **BOLD** | **Loc.** |
| --- | --- | --- | --- | --- |
| ***Akodon azarae*** | | | | |
| UFPB7388 | A17 | GU938880 | 350-12 | 2 |
| UFPB7389 | JR328 | GU938989 | 461-12 | 10 |
| UFPB7390 | JR329 | GU938990 | 462-12 | 10 |
| LCEUFRGS1787 | 1787 | GU938911 | 598-12 | - |
| LCEUFRGS1788 | 1788 | GU938912 | 599-12 | - |
| ***Akodon montensis*** | | | | |
| UFRGS | DG21 | GU938873 | 403-12 | 6 |
| UFRGS | DG22 | GU938874 | 404-12 | 6 |
| UFRGS | DG23 | GU938875 | 405-12 | 6 |
| FURB9826 | - | GU938932 | 476-12 | 13 |
| FURB9595 | - | GU938933 | 417-12 | 13 |
| UFPB7391 | JR197 | GU938920 | 439-12 | 7 |
| UFPB7392 | JR198 | GU938921 | 440-12 | 7 |
| UFPB7393 | JR199 | GU938923 | 441-12 | 7 |
| UFPB7394 | JR200 | GU938922 | 442-12 | 7 |
| UFPB7395 | JR203 | GU938924 | 444-12 | 7 |
| UFPB7396 | JR264 | GU938925 | 455-12 | 7 |
| UFPB7397 | JR461 | GU938919 | 484-12 | 7 |
| UFPB7398 | JR535 | GU938931 | 488-12 | 7 |
| UFPB7399 | JR335 | GU938926 | 464-12 | 5 |
| UFPB7400 | JR336 | GU938927 | 465-12 | 5 |
| UFPB7401 | JR338 | GU938928 | 467-12 | 10 |
| UFPB7402 | JR386 | GU938929 | 473-12 | 8 |
| UFPB7403 | JR389 | GU938930 | 474-12 | 8 |
| UFPB7404 | JR407 | GU938934 | 476-12 | 9 |
| UFPB7405 | PCE24 | GU938913 | 551-12 | 3 |
| ***Akodon paranaensis*** | | | | |
| FURB6844 | - | GU938992 | 410-12 | 11 |
| FURB6963 | - | GU938993 | 413-12 | 11 |
| UFPB7406 | JR411 | GU938991 | 478-12 | 9 |
| ***Calomys cerqueirai*** | | | | |
| MNRJ71971 | LBCE5556 | GU938940 | 526-12 | 16 |
| ***Calomys expulsus*** | | | | |
| LMUERJ-LG408 | LG408 | GU938937 | 528-12 | 21 |
| LMUERJ-LG443 | LG443 | GU938936 | 529-12 | 17 |
| MNRJ63373 | LBCE1548 | GU938938 | 513-12 | 20 |
| MNRJ63372 | LBCE1547 | GU938939 | 512-12 | 20 |
| MNRJ71962 | CRB2374 | GU938941 | 391-12 | 10 |
| LBPMSR CRB2732 | CRB2732 | GU938942 | 393-12 | 19 |
| LBPMSR CRB2733 | CRB2733 | GU938943 | 394-12 | 19 |
| ***Calomys tener*** | | | | |
| LDCM-AB6 | AB6 | GU939000 | 360-12 | 15 |
| LDCM-AB10 | AB10 | GU939002 | 358-12 | 15 |
| LDCM-AB519 | AB519 | GU939001 | 359-12 | 15 |
| MNRJ67075 | CRB2382 | GU938935 | 392-12 | 18 |
| UFPB7407 | JR405 | GU938944 | 475-12 | 2 |
| ***Delomys dorsalis*** | | | | |
| UFPB7576 | DD2 | GU938903 | 395-12 | 12 |
| UFPB7577 | DD3 | GU938902 | 396-12 | 12 |
| UFPB7578 | DD40 | GU938901 | 397-12 | 12 |
| UFPB7408 | JR207 | GU938948 | 446-12 | 7 |
| UFPB7409 | JR224 | GU938946 | 224-12 | 7 |
| UFPB7410 | JR229 | GU938907 | 451-12 | 7 |
| UFPB7411 | JR230 | GU938904 | 452-12 | 7 |
| UFPB7412 | JR290 | GU938900 | 457-12 | 7 |
| UFPB7413 | JR349 | GU938905 | 470-12 | 7 |
| UFPB7414 | JR536 | GU938945 | 489-12 | 7 |
| UFPB7415 | JR538 | GU938906 | 490-12 | 7 |
| UFPB7416 | JR527 | GU938949 | 487-12 | 7 |
| ***Delomys sublineatus*** | | | | |
| FURB9331 | - | GU938951 | 415-12 | 13 |
| FURB9950 | - | GU938952 | 428-12 | 13 |
| ***Deltamys kempi*** | | | | |
| MNHN6194 | PCE05 | GU938917 | 546-12 | 1 |
| MNHN6196 | PCE06 | GU938918 | 547-12 | 1 |
| MNHN6197 | PCE11 | GU938914 | 548-12 | 1 |
| MNHN7639 | PCE12 | GU938915 | 549-12 | 1 |
| MHHN7640 | PCE13 | GU938916 | 550-12 | 1 |
| ***Deltamys* sp. n.** | | | | |
| UFPB7589 | A7 | GU938879 | 357-12 | 12 |
| UFPB7590 | A10 | GU938996 | 349-12 | 12 |
| MCP1658 | A24 | GU938881 | 351-12 | 12 |
| UFPB7591 | A26 | GU938882 | 353-12 | 12 |
| MNRJ68940 | A25 | GU938883 | 352-12 | 12 |
| UFPB7592 | A29 | GU938884 | 354-12 | 12 |
| UFPB7593 | A33 | GU938994 | 355-12 | 12 |
| UFPB7594 | A43 | GU938995 | 356-12 | 12 |
| ***Drymoreomys albimaculatus*** | | | | |
| FURB9792 |  | GU938885 | 670-12 | 13 |
| ***Euryoryzomys russatus*** | | | | |
| FURB9409 | - | GU938955 | 416-12 | 13 |
| FURB9954 | - | GU938947 | 429-12 | 13 |
| FURB9695 | - | GU938960 | 419-12 | 13 |
| FURB9773 | - | GU938958 | 421-12 | 13 |
| FURB9994 | - | GU938950 | 430-12 | 13 |
| UFPB7417 | JR194 | GU938959 | 437-12 | 7 |
| UFPB7418 | JR205 | GU938954 | 445-12 | 7 |
| UFPB7419 | JR208 | GU938957 | 447-12 | 7 |
| UFPB7420 | JR281 | GU938956 | 456-12 | 7 |
| UFPB7421 | JR297 | GU938896 | 458-12 | 7 |
| UFPB7422 | JR298 | GU938897 | 459-12 | 7 |
| UFPB7423 | JR299 | GU938895 | 460-12 | 7 |
| ***Juliomys pictipes*** | | | | |
| FURB9735 | - | GU938963 | 420-12 | 13 |
| ***Mus musculus*** | | | | |
| FURB9667 | - | GU938964 | - | 13 |
| ***Necromys lasiuru****s* | | | | |
| FURB6859 |  | GU938998 | - | 13 |
| UFPB7424 | JR346 | GU938997 | 469-12 | 4 |
| ***Nectomys squamipes*** | | | | |
| FURB9905 | - | GU938967 | 425-12 | 13 |
| FURB9790 | - | GU938968 | 422-12 | 13 |
| ***Oligoryzomys flavescens*** | | | | |
| UFPB7447 | DG15 | GU938891 | 402-12 | 6 |
| UFPB7425 | JR202 | GU938986 | 443-12 | 7 |
| UFPB7426 | JR209 | GU938985 | 448-12 | 7 |
| UFPB7427 | JR210 | GU938987 | 449-12 | 7 |
| UFPB7428 | JR258 | GU938893 | 453-12 | 7 |
| UFPB7429 | JR259 | GU938894 | 454-12 | 7 |
| UFPB7430 | JR467 | GU938892 | 485-12 | 7 |
| UFPB7431 | JR332 | GU938988 | 463-12 | 2 |
| ***Oligoryzomys nigripes*** | | | | |
| FURB6884 | - | GU938971 | 412-12 | 11 |
| FURB9600 | - | GU938969 | 418-12 | 13 |
| UFPB7443 | DG00 | GU938877 | 398-12 | 6 |
| UFPB7444 | DG01 | GU938886 | 399-12 | 6 |
| UFPB7445 | DG05 | GU938878 | 400-12 | 6 |
| UFPB7446 | DG06 | GU938876 | 401-12 | 6 |
| UFPB7432 | JR337 | GU938976 | 466-12 | 5 |
| UFPB7433 | JR339 | GU938979 | 468-12 | 10 |
| UFPB7434 | JR409 | GU938975 | 477-12 | 9 |
| UFPB7435 | JR363 | GU938977 | 471-12 | 7 |
| UFPB7436 | JR425 | GU938888 | 479-12 | 7 |
| UFPB7437 | JR426 | GU938889 | 480-12 | 7 |
| UFPB7438 | JR457 | GU938890 | 481-12 | 7 |
| UFPB7439 | JR458 | GU938898 | 482-12 | 7 |
| UFPB7440 | JR459 | GU938899 | 483-12 | 7 |
| UFPB7441 | JR518 | GU938887 | 486-12 | 7 |
| UFPB7442 | OLN31 | GU938970 | 534-12 | 12 |
| UFPB7579 | ON1 | GU938978 | 535-12 | 12 |
| UFPB7580 | OPN52 | GU938980 | 536-12 | 12 |
| UFPB7581 | OPN55 | GU938981 | 537-12 | 12 |
| UFPB7582 | OPN57 | GU938984 | 538-12 | 12 |
| UFPB7583 | OPN58 | GU938974 | 539-12 | 12 |
| UFPB7584 | OPN59 | GU938973 | 540-12 | 12 |
| UFPB7585 | OPN61 | GU938972 | 541-12 | 12 |
| UFPB7586 | OPN64 | GU938982 | 542-12 | 12 |
| UFPB7587 | OPN87 | GU938983 | 543-12 | 12 |
| ***Rhagomys rufescens*** | | | | |
| FURB9908 | - | GU938965 | 426-12 | 13 |
| FURB9921 | - | GU938966 | 427-12 | 13 |
| ***Sooretamys angouya*** | | | | |
| FURB9793 | - | GU938961 | 423-12 | 13 |
| UFPB7448 | JR196 | GU938962 | 438-12 | 7 |
| ***Thaptomys nigrita*** | | | | |
| UFPB7449 | JR372 | GU938909 | 472-12 | 8 |
| FURB6559 | - | GU938910 | 409-12 | 13 |
| UFPB7588 | TN3 | GU938908 | 619-12 | 12 |
| ***Unknown*** | | | | |
| LCEUFRGS1007 | 1007 | GU938999 | - | 14 |
| LCEUFRGS9629 | 9629 | GU938953 | 671-12 | - |

Footnote: Columns correspond to collection number (Sample ID), field number (Field ID), GenBank Accession (GenBank), BOLD number of project ROSBR (BOLD) and locality numbers corresponding to Figure 1 (Loc.). Collection abbreviations: FURB: Fundação Universidade Regional de Blumenau; LBPMSR: Laboratório de Biologia e Parasitologia de Mamíferos Silvestres Reservatórios of the Fundação Oswaldo Cruz; LCE-UFRGS:Laboratório de Citogenética e Evolução of Universidade Federal do Rio Grande do Sul; LDCM: Laboratório de Diversidade e Conservação de Mamíferos of the Universidade de São Paulo; LMUERJ: Laboratório de Mastozoologia of the Universidade do Estado do Rio de Janeiro; MCP: Museu de Ciência e Tecnologia of the Pontificia Universidade Católica do Rio Grande do Sul; MNRJ: Museu Nacional - Universidade Federal do Rio de Janeiro; MNHN: Museo Nacional de Historia Natural de Uruguay; UFPB –Universidade Federal da Paraíba.
