# Supplementary material for: DNA Barcoding of Sigmodontine Rodents: Identifying Wildlife Reservoirs of Zoonoses
Source: PLoS One. 2013 Nov 11;8(11):e80282. doi: 10.1371/journal.pone.0080282 (PMC3823626; doi:10.1371/journal.pone.0080282)
Supplement: Table S2 — Parameter estimates for GTR+I+G model from Maximum likelihood (ML) and Bayesian inference (BI). (DOC) [file pone.0080282.s003.doc]

**Table S2.** Parameter estimates for GTR+I+G model from Maximum likelihood (ML) and Bayesian inference (BI).

| **Model** | **ML** | **BI** (x; σ^2^) |
| --- | --- | --- |
| P_inv_ | 0.3380 | 0.3597; 0.000388 |
| Ts/Tv ratio | 4.6950 | 6.0769; 0.104496 |
| f(A) | 0.2854 | 0.3173; 0.000152 |
| f(C) | 0.2600 | 0.2793; 0.000074 |
| f(G) | 0.1609 | 0.0734; 0.000014 |
| f(T) | 0.2938 | 0.3300; 0.000090 |
| α | 0.7120 | 0.5215; 0.000390 |
| Ln Likelihood | -14115.5 | -14225 |

Footnote: P_inv_: proportion of invariant sites. Ts/T_V_: Transition/Transversion ratio. f (N): nucleotide frequency. α: gamma distribution shape parameter value. Mean and variance are represented by x and σ^2^, respectively.
